# Reinforcement of Rubber Magnetic Composites with Zinc Salts of Acrylic and Methacrylic Acids

**DOI:** 10.3390/ma11112161

**Published:** 2018-11-01

**Authors:** Ján Kruželák, Viera Karlíková, Rastislav Dosoudil, Katarína Tomanová, Ivan Hudec

**Affiliations:** 1 Department of Plastics, Rubber and Fibres, Faculty of Chemical and Food Technology, Slovak University of Technology in Bratislava, Radlinského 9, 812 37 Bratislava, Slovakia; katarina.tomanova@stuba.sk (K.T.); ivan.hudec@stuba.sk (I.H.); 2 Polymer Institute, Slovak Academy of Sciences, Dúbravská cesta 9, 845 41 Bratislava, Slovakia; viera.karlikova@savba.sk; 3 Department of Electromagnetic Theory, Faculty of Electrical Engineering and Information Technology, Slovak University of Technology in Bratislava, Iľkovičova 3, 812 19 Bratislava, Slovakia; rastislav.dosodil@stuba.sk

**Keywords:** strontium ferrite, rubber composites, peroxide cross-linking, co-agents

## Abstract

Strontium ferrite was compounded with acrylonitrile butadiene rubber to prepare rubber magnetic composites. For cross-linking of the prepared materials, peroxide curing systems consisting of dicumyl peroxide as curing agent and zinc salts of acrylic and methacrylic acids as co-agents were used. The amount of strontium ferrite was kept constant in all experiments, while the main objective of the work was to investigate the composition of curing system and both types of co-agents on the cross-linking, physical-mechanical, dynamic and magnetic properties of the rubber magnets. The results showed that the change in composition of curing system has significant influence on cross-link density and properties of the tested composite materials. With an increasing amount of zinc based co-agents, significant improvement of tensile strength was achieved. The application of zinc based co-agents in peroxide vulcanization of rubber magnetic composites leads to the preparation of rubber magnets with not only good magnetic properties, but also with improved physical-mechanical characteristics.

## 1. Introduction

Rubber magnetic composites rank among the developing class of smart materials that have already found utilization in many special applications, in microwave and radar technology, motor parts, magnetic imaging, memo holders, as well as in form of sensors or shielding materials of electromagnetic and magnetic fields [1,2,3,4]. Rubber magnetic composites combine a unique pattern of suitable magnetic and elastic properties; they are characterized by easy adaptability, excellent flexibility, and corrosion resistance. They can be coiled, curved, and shaped with the retention of their magnetic properties.

The optimal elastic and tensile properties of engineering rubber materials are obtained during the process of vulcanization which is simply termed as curing. The substance of vulcanization is the formation of different types of chemical linkages between elastomer chain segments that contribute to the generation of the three-dimensional network structure within the rubber matrix [5,6]. A lot of vulcanization systems have been introduced in cross-linking of elastomers, which include sulfur based curing systems, organic peroxides, phenolic resins, metal oxides, and quinones, etc. Among them, sulfur and peroxide vulcanization systems are the most frequently used. 

Sulfur curing is the oldest process that is used for cross-linking of diene unsaturated rubbers. During this process, rubber macromolecules are linked together with different types of sulfur based cross-links, i.e., mono-, di- and polysulfidic cross-links. It is well known that sulfur vulcanization is very complex and intricate process and for optimal cross-linking, sulfur is used in combination with accelerators and activators. Sulfur cured vulcanizates generally show good tensile and elastic behavior, high tear and tensile strength, but limited heat ageing stability and weak resistance to thermo-oxidative ageing [7,8,9]. 

The application of organic peroxides in cross-linking of rubber compounds leads to the formation of carbon-carbon linkages between rubber chain segments [10,11]. Carbon-carbon cross-links have higher bonding energy in comparison with sulfur based cross-links. Therefore, the main features of peroxide cross-linked elastomers are high thermal stability and excellent resistance to ageing. Low compression set, good electrical properties, or the simple formulation of rubber compounds are the next advantages of peroxide cured elastomers [12,13,14]. Though, there are also some drawbacks when compared to sulfur curing systems, as worse elastic and dynamic properties, worse tensile strength and lower abrasion resistance of vulcanizates [8,15]. 

To improve the cross-linking of rubbers with organic peroxides, the multifunctional low molecular weight monomers, the so called co-agents are often applied [16,17,18,19,20]. Co-agents exhibit high reactivity towards free radicals and by homopolymerization or grafting onto rubber chains they contribute to the increase of curing efficiency and cross-link density of the rubber articles. This subsequently leads to the improvement of treatability and properties of the peroxide cured elastomers [21,22,23]. 

A lot of scientific works have been devoted to the preparation of rubber composites with incorporated magnetic fillers [24,25,26,27,28]. The results revealed that the application of magnetic powdery fillers into various elastomer matrices imparts magnetic properties to the composites significantly; however, their physical-mechanical characteristics were usually worsened [26,27,28]. The reason might be attributed to the weak compatibility and adhesion between rubber matrices and magnetic fillers. Therefore, the main objective of the work was to prepare rubber magnets not only with adequate magnetic characteristics, but mainly with applicable physical-mechanical properties. Dicumyl peroxide was used in combination with zinc acrylate or zinc methacrylate for the cross-linking of rubber magnetic composites. It has been reported that zinc based co-agents exhibit strong adhesion to polar materials [8,14,17]. As ferrites are also polar materials, the selection of co-agents was targeted to improve the adhesion and compatibility on the interphase filler-rubber. The achieved results revealed that by suitable combination of organic peroxide with co-agents, it is possible to prepare rubber composites not only with good magnetic properties, but as well with enhanced applicable characteristics.

## 2. Experimental

### 2.1. Materials

**Figure 1:**
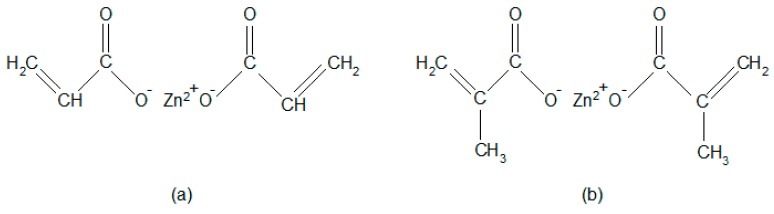
The structural formulas of (**a**) zinc acrylate and (**b**) zinc methacrylate.

Acrylonitrile butadiene rubber NBR with trade mark SKN 3345 and 31–35% content of acrylonitrile was supplied by Sibur International, Moscow, Russia. As magnetic filler, strontium ferrite SrFe_12_O_19_, type FD 8/24 (Magnety, Světlá Hora, Czech Republic) was applied. Dicumyl peroxide DCP (Merck Schuchardt OHG, Hohenbrunn, Germany) was used as a curing agent and acrylic acid zinc salt ZDA (zinc acrylate, Sigma-Aldrich, St. Louis, Missouri, MO, USA) and methacrylic acid zinc salt ZDMA (zinc methacrylate, Sigma-Aldrich, St. Louis, Missouri, MO, USA) were used as co-agents. The structural formulas of both co-agents are illustrated in Figure 1.

### 2.2. Methods

#### 2.2.1. Determinstion of Structural and Magnetic Characteristics of Ferrite

Total porosity, specific surface area, and total volumes of pores were determined by using the method of mercury porosimetry. The measurements were performed by applying Porosimeter 1500 (Carlo Erba, Milan, Italy). Pores up to the size of 5 nm are possible to be determined at a maximal mercury press of 150 MPa. Density of ferrite was provided by the manufacturer. Magnetic characteristics of ferrite were determined by using Magnetometer TVM-1 (Vúzort, Praha, Czech Republic). The structural and magnetic characteristics of magnetic filler are referred in Table 1.

#### 2.2.2. Preparation and Curing of Rubber Compounds

Compounding of rubber formulations was performed in a chamber of laboratory equipment Brabender (B 50, Brabender GmbH & Co.KG, Duisburg, Germany) at 90 °C and rotor speed of 50 rpm. First, NBR was plasticated for 2.5 min, then ferrite was added. After next 2 min, ZDA or ZDMA were introduced. The total time of first step mixing was 9 min at 90 °C. DCP was added in the second step and compounded for 4 min. After that, the compounds were homogenized and shaped in two roll calender. 

In the first part of the study, rubber magnetic composites were prepared by the incorporation of a constant amount of DCP into NBR based rubber matrix, while the main aim was to investigate the amount of co-agents ZDMA or ZDA on the cross-linking and properties of the prepared materials. The amount of strontium ferrite was also kept constant in all composites. Table 2 summarizes the composition of rubber magnets that were tested in the first part of the research. Phr stands for parts per hundred rubber.

The main aim of the second part of the study was to examine the amount of DCP on the behavior of rubber magnets with constant level of co-agents and magnetic filler. The composition of rubber compounds with different amount of DCP is mentioned in Table 3.

The cross-linking of rubber compounds was carried out for the optimum vulcanization time at 160 °C and 15 MPa by using a hydraulic press Fontijne (LabEcon 300, Fontijne Presses, Barendrecht, The Netherlands). Finally, thin rubber sheets with size 15 cm × 15 cm and thickness of 2 mm were obtained.

#### 2.2.3. Determination of Cross-Link Density of Composites

The cross-link density *ν* was determined based on equilibrium swelling of composite samples in acetone. The weighted dried samples were placed into acetone in which they swelled within time at a laboratory temperature. The weight of samples was measured every hour until the equilibrium swelling was reached. The Flory-Rehner equation, as modified by Krause [29], was then used to calculate the cross-link density:

(1) $$\nu =-\frac{V_{r0}}{V_{S}}\frac{\ln(1-V_{r})+V_{r}+\chi V_{r}^{2}}{V_{r}^{1/3}V_{r0}{}^{2/3}-0.5V_{r}},$$

*ν*—Cross-link density (mol/cm^3^)
*V_r_*_0_—Volume fraction of rubber in equilibrium swelling sample of vulcanizate in absence of fillers
*V_r_*—Volume fraction of rubber in equilibrium swelling sample of filled vulcanizate
*V_S_*—Molar volume of solvent (for acetone = 73.52 cm^3^/mol)
*χ*—Huggins interaction parameter (for NBR-acetone *χ* = 0.3692)

#### 2.2.4. Determination of Physical-Mechanical Properties

Tensile strength, elongation at break and modulus of composites were evaluated by using Zwick Roell tearing machine (Z 2.5, ZwickRoell GmbH & Co.KG, Ulm, Germany) at a cross-head speed of 500 mm/min following the valid technical standards. Dumbbell-shaped test specimens with length 80 mm, thickness 2 mm, and width 6.4 mm were used for measurements. Durometer in Shore A scale was used to determine hardness of composites.

#### 2.2.5. Evaluation of Dynamical-Mechanical Properties

Dynamical-mechanical behavior of composites was tested by introducing a dynamical-mechanical analyzer (DMTA MkIII, Anton Paar GmbH, Graz, Austria). The composites were analyzed at a frequency 10 Hz in a tensile mode. The measurements were carried out in temperature range from −60 °C to 60 °C at amplitude of dynamic deformation 64 μm and a static force 0.2 N.

#### 2.2.6. Determination of Magnetic Characteristics

Magnetometer (TVM−1, Vúzort, Praha, Czech Republic) was used to determine magnetic characteristics of composites. The measurements were performed at a maximum coercive intensity of magnetic field 750 kA/m and at a laboratory temperature. Scattering magnetic flux Φ induced by magnetic vibrating specimen is scanned by applying the magnetic induction method. The samples for the determination of magnetic characteristics were prism shaped (8 mm × 4 mm × 2 mm). 

#### 2.2.7. Microscopic Analysis of Composites

The surface morphology and microstructure of composite materials were examined by using a scanning electron microscope (JEOL JSM-7500F, JEOL, Ltd., Tokyo, Japan). The samples were first cooled down in a liquid nitrogen under the glass transition temperature and then fractured into small fragments with surface area of 3 mm × 2 mm. The fractured surface was covered with a thin layer of gold and then put under the microscope objective.

## 3. Results and Discussions

### 3.1. Influence of Co-Agents Content on Cross-Linking and Properties of Rubber Magnets Cured with Constant Amount of Dicumyl Peroxide

In the first part of the study, rubber composites filled with constant level of magnetic filler—50 phr were cured with constant amount of dicumyl peroxide—1 phr and the goal was to examine the content of co-agents ZDA and ZDMA on the cross-link density and properties of the composites. Both co-agents were incorporated into the rubber formulations in the amount ranging from 10 to 50 phr. 

#### 3.1.1. Cross-Link Density and Physical-Mechanical Properties

**Figure 2:**
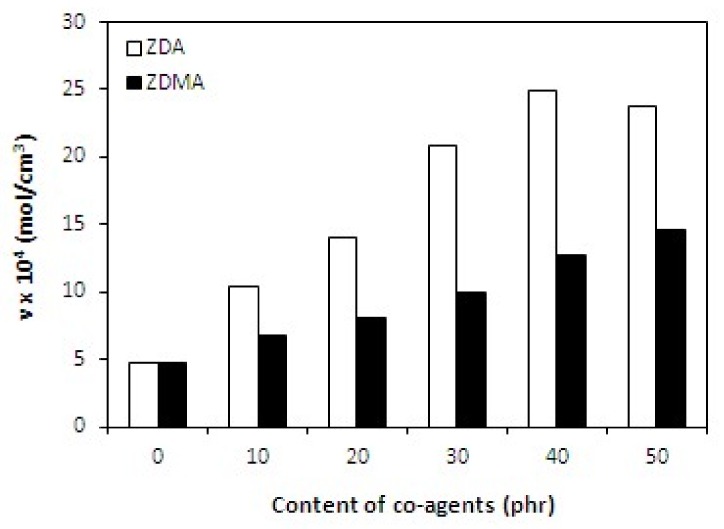
Influence of co-agents content on cross-link density *ν* of composites.

The cross-link density is an essential structural parameter of all cured rubber compounds, which significantly influences their properties. The cross-link density of composites was determined based on swelling of samples in acetone. When equilibrium swelling degree was achieved, the Flory-Rehner formula that was modified by Krause was used to compute the cross-link density. The achieved results showed that the co-agents have positive influence on the cross-link density which was found to significantly increase with increasing amount of both type co-agents. It also becomes clearly obvious from Figure 2 that the cross-link density of composites cured with dicumyl peroxide and zinc acrylate was much higher in comparison with the composites that were cured with dicumyl peroxide and zinc methacrylate.

**Figure 3:**
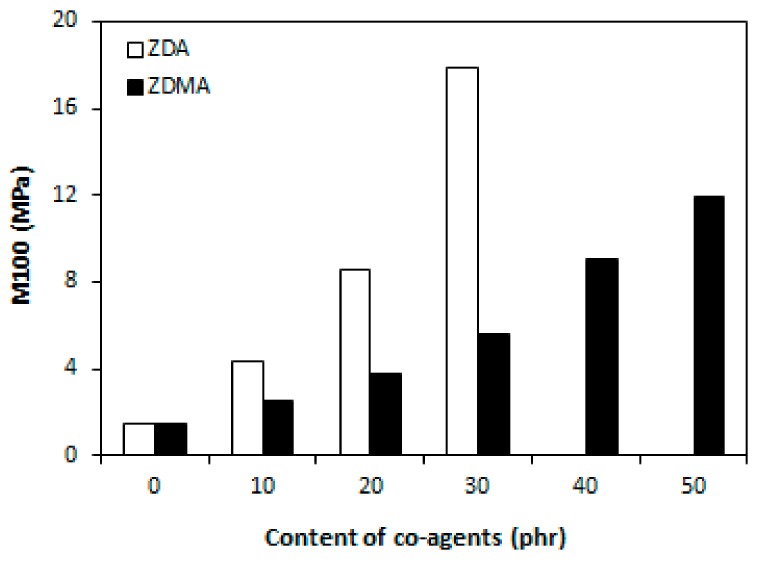
Influence of co-agents content on modulus M100 of composites.

**Figure 4:**
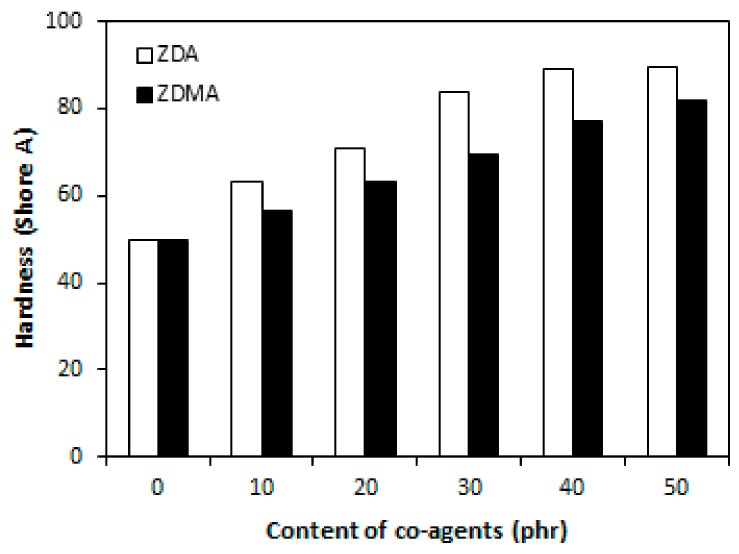
Influence of co-agents content on hardness of composites.

An increasing trend of cross-link density was subsequently reflected in improvement of modulus M100 and hardness of composites with increasing amount of co-agents (Figure 3 and Figure 4). Higher modulus and hardness of composites that were cured with DCP and ZDA as well describe close relation between dependencies of cross-link density and both characteristics. The modulus M100 of composites with 40 and 50 phr of ZDA was not possible to measure, because these composites were ruptured before reaching 100% deformation. 

**Figure 5:**
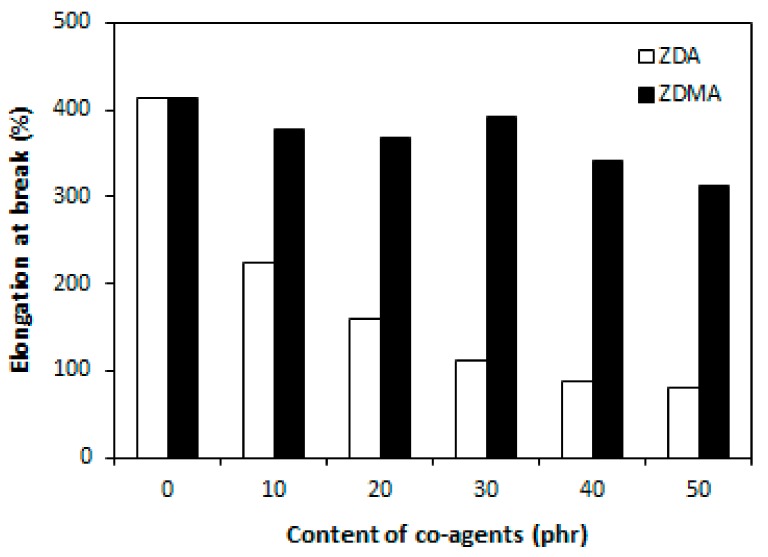
Influence of co-agents content on elongation at break of composites.

Higher cross-link density of composites that were cured with DCP and ZDA was, on the other hand, responsible for much lower elongation at break and a much more significant decrease of elongation at break with increasing amount of ZDA (Figure 5). The elongation at break of the composite with maximum ZDA content decreased in more than 300% in comparison to the reference composite that was cured only with peroxide (from above 400% for the composite cured in absence of co-agent to less than 100% for the composite with 50 phr of zinc acrylate). As also shown Figure 5, the decrease of elongation at break of composites cured with DCP and ZDMA was not very significant and even at the maximum ZDMA content, the elongation at break was of this composite overcame 300%.

**Figure 6:**
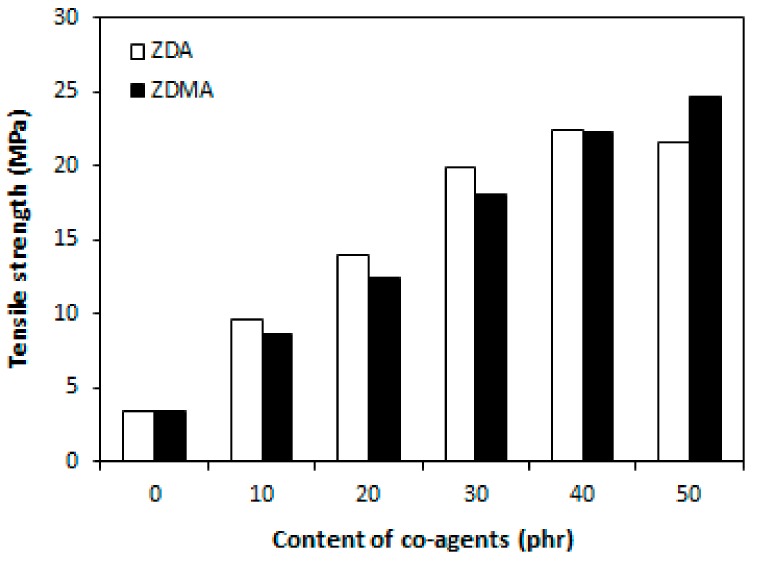
Influence of co-agents content on tensile strength of composites.

From the graphical dependence of tensile strength on co-agents content (Figure 6), it is possible to see that the tensile strength of both type composites was significantly improved with increasing the amount of both zinc acrylate and zinc methacrylate. The tensile strength of composites increased from less than 5 MPa to almost 25 MPa when the amount of co-agents increased from 0 up to 50 phr. This represents a remarkable increase in more than 20 MPa. Except for the composites with maximum co-agents content, slightly higher tensile strength were found to achieve composites that were cured with DCP and ZDA. 

**Figure 7:**
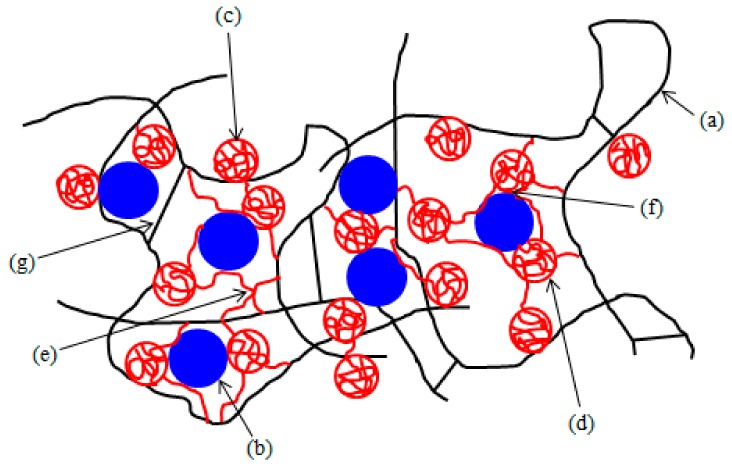
Schematic representation of the micro-structure model of rubber magnetic composites cured in the presence of peroxide and zinc acrylate or zinc methacrylate: (**a**) rubber chains; (**b**) particles of ferrite; (**c**) polymerized molecules of co-agents physically adsorbed onto rubber chains; (**d**) polymerized molecules of co-agents chemically grafted onto rubber chains; (**e**) ionic physical cross-links formed from co-agents; (**f**) physical interactions between particles of ferrite and polymerized molecules of co-agents; and, (**g**) covalent carbon-carbon bonds.

The reason for the increase of cross-link density and improvement of tensile strength can be attributed to the presence of co-agents in rubber formulations with magnetic filler. It has been reported that the in situ radical polymerization of zinc based co-agents takes place during the vulcanization process of rubber compounds with organic peroxides [30,31,32,33]. Polymerized molecules of ZDA and ZDMA tend to aggregate to form spherical nanoparticles that can be chemically grafted or physically adsorbed onto elastomer chains. Thus, they contribute to the formation of physical and chemical couplings in the rubber matrix. Moreover, due to the presence of zinc ions in ZDA and ZDMA, co-agent molecules can also form ionic clusters through the static electronic attractions. Ionic clusters can also be considered as the type of physical cross-links in the rubber matrix [30,34,35,36,37]. It can be stated that ZDA and ZDMA as co-agents in peroxide curing reinforce the rubber matrix [38,39,40]. Secondly, it has been demonstrated that zinc based co-agents exhibit strong adhesion to polar materials [41,42]. Ferrites are chemical compounds of metal oxides. Due to the presence of metal and oxygen ions they belong to the polar materials [43,44]. Zinc based co-agents increase the polarity of the rubber matrix by grafting onto polymer chains on one hand, and they physically interact with particles of magnetic filler on the other hand. Based upon the mentioned aspects, it can be stated that by physical adsorption and chemical grafting of co-agents onto rubber chains and their strong physical interactions with strontium ferrite, ZDA and ZDMA contribute to the improvement of adhesion and compatibility on the interphase filler—rubber. Subsequently, the overall improvement of physical-mechanical properties of rubber magnetic composites was achieved. A schematic representation of the micro-structure model of rubber magnets cured in the presence of peroxide and zinc based co-agents is graphically illustrated in Figure 7. Although the reaction mechanisms of both type co-agents seem to be similar, based upon determination of cross-link density and physical-mechanical properties of tested composites it might be stated that ZDMA contributes mainly to the formation of ionic clusters and physical-cross-links in the rubber matrix with less adhesion to magnetic filler. This presumption is supported by lower chemical cross-link density (Figure 2) and higher elongation at break of the corresponding composites (Figure 5). Composite systems with this type of morphology exhibit the capability of stress relaxation, due to rubber chain slippage on the surface of ionic clusters and nanoparticles and the recovery of ionic couplings upon external deformation of the composites [42,45]. This mechanism is suggested to be similar to that proposed for the rubber network structure formed from sulfidic cross-links [42]. By contrast, ZDA contributes mainly to the formation of chemical cross-links in the rubber matrix, perhaps due to the absence of methyl groups that could act as steric hindrances against creation of chemical couplings with the rubber matrix (Figure 1). On the other hand, ZDA exhibits stronger adhesion to magnetic filler. This presumption is supported by higher tensile strength of composites that were cured with DCP and ZDA (Figure 6), as good adhesion and compatibility between the filler and the rubber on the interphase are the most important factors of the reinforcement of rubber composites [14,46,47].

#### 3.1.2. Microscopic Analysis

**Figure 8:**
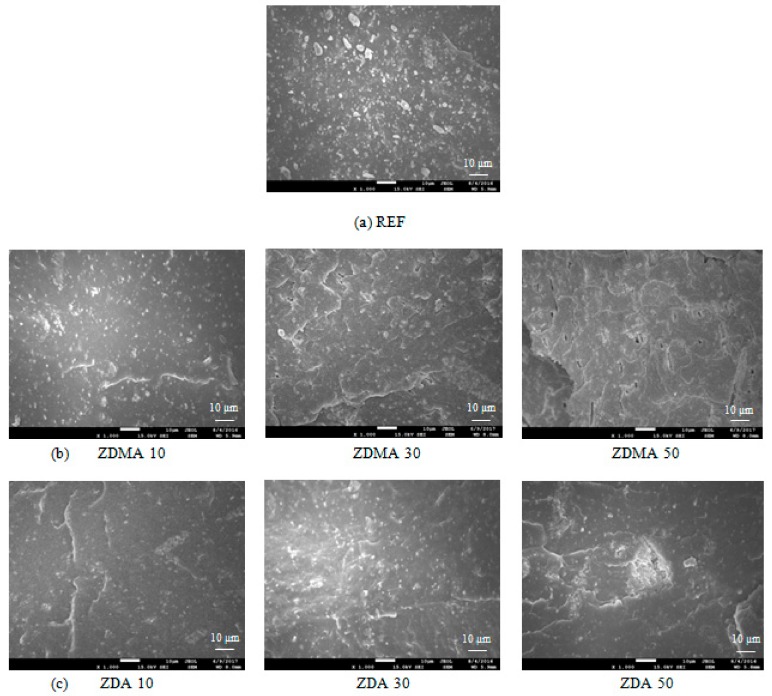
SEM images of composites: (**a**) reference composite cured only with peroxide; (**b**) composites cured with peroxide and zinc methacrylate; and, (**c**) composites cured with peroxide and zinc acrylate.

SEM analysis confirmed the mentioned statements showing that the least homogenous structure showed reference composite cured only with peroxide. From Figure 8a, it is possible to observe the aggregates of strontium ferrite that seems to be not well dispersed in the elastomer matrix. The incorporation of co-agents leads to the better dispersion of magnetic filler (Figure 8b,c). This can be attributed to a higher viscosity of the rubber matrix and thus higher shear rates during compounding on one hand, and to good adhesion of co-agents to magnetic filler on the other hand. It is also possible to observe from SEM images that the compatibility and homogeneity between ferrite and the rubber matrix is higher in the case of composite materials that were cured with DCP and ZDA (Figure 8c). In the case of composites cured with DCP and ZDMA, there is more evidently visible the presence of microcavities and defects on the filler—rubber interphase (Figure 8b). This seems to be a strong evidence confirming the supposition that zinc acrylate shows better adhesion to strontium ferrite and thus it contributes to a stronger improvement of adhesion and compatibility between the filler and the rubber.

#### 3.1.3. Dynamic-Mechanical Analysis

**Figure 9:**
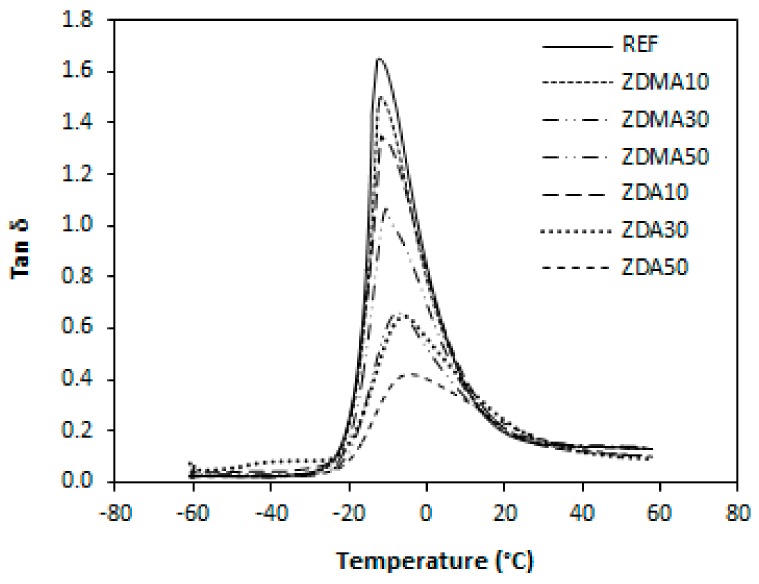
Variations of tan δ of reference composite cured only with peroxide and composites cured with different content of co-agents.

Figure 9 illustrates the variation of tan δ for rubber magnets in dependence on the type and amount of co-agents. The peak maximum represents the T_g_ and it becomes clearly evident that the glass transition temperature of the composites is dependent on their cross-link density as also possible to see in Table 4. The lowest T_g_ was found to have composite cured only with peroxide with the lowest degree of cross-linking. With increasing amount of co-agents, the cross-link density increased causing lower molecular mobility, and thus the T_g_ shifted to higher temperatures. When comparing both type composites, lower T_g_ exhibited composites cured with DCP and ZDMA with lower cross-link density when compared to the equivalent composites cured with DCP and ZDA. 

#### 3.1.4. Magnetic Characteristics

It has been described in scientific studies that the incorporation of ferrite powdery fillers into different rubber compounds gives magnetic properties to composites significantly [24,26,27,48,49,50]. As in the present work, the content of strontium ferrite was kept on a constant level in tested composites; the main objective was to investigate the change in composition of curing system on the magnetic characteristics. The coercive intensity of magnetic field *H_c_*, or shortly coercivity and the remanent magnetic induction *B_r_* characterize all permanent magnets. Remanent magnetic induction means the value of remaining magnetization in the magnet after magnetizing it in the presence of external magnetic field. The coercivity means the energy of magnetic field that is required to remove the remanent magnetic induction from the magnet. 

**Figure 10:**
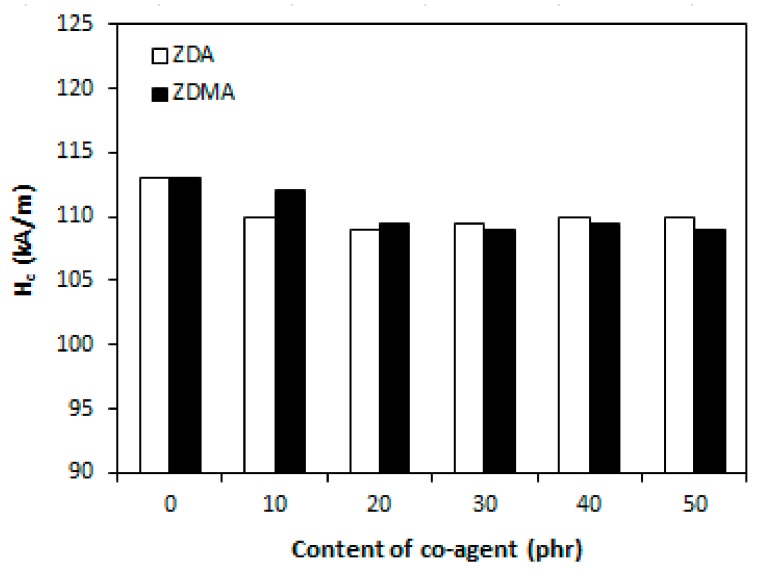
Influence of co-agents content on coercivity *H_c_* of composites.

**Figure 11:**
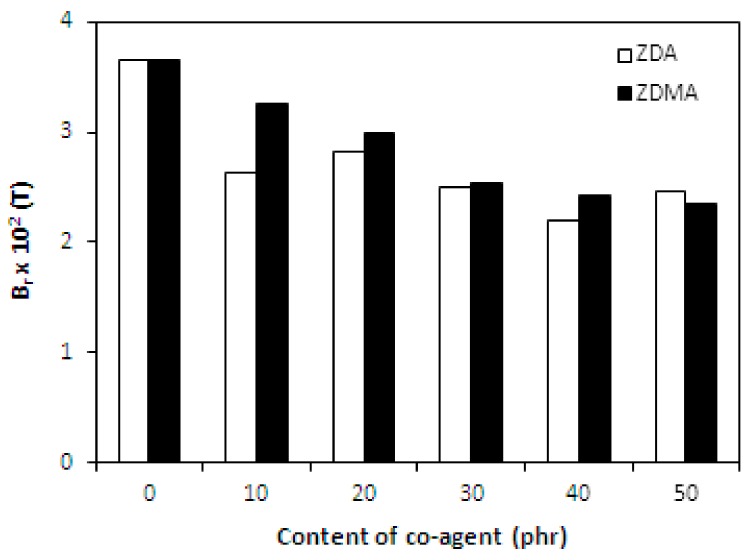
Influence of co-agents content on remanent magnetic induction *B_r_* of composites.

From Figure 10 it is possible to observe that the values of coercivity for both type composites fluctuated only in a low range of experimental values, independently on the type and amount of co-agents. On the other hand, the presence of co-agents leads to a slight deterioration of the remanent magnetic induction (Figure 11). The possible explanation might be the presence of non-magnetic zinc ions from co-agent molecules in the rubber matrix, which could as a shield against magnetic flow through the composites. The higher was the amount of co-agents, the higher was the amount of zinc ions, and the more was shielded the magnetic flow through the composites. Thus, the remanent magnetic induction of composite materials decreased. 

### 3.2. Influence of Peroxide Content on Cross-Link Density and Properties of Rubber Magnetic Composites Cured with Constant Amount of Co-Agents

The results that were achieved in the first part of the study revealed that the presence of zinc based co-agents in peroxide curing of rubber magnetic composites has significant influence on cross-link density, physical-mechanical, and dynamic properties. By contrast, there was recorded only a slight influence of co-agents on magnetic characteristics. However, the conversion of zinc based co-agents during the in situ reaction in the rubber matrix does not reach 100%. The residual monomers from co-agents exist in the composites in the form of micro-level dispersion [30,34,35]. Several factors determine the conversion of zinc based co-agents in the rubber matrix among which the presence of free radicals is the most important [30,35]. Therefore, the main objective of the second part of the study was to investigate the amount of dicumyl peroxide on the properties of rubber magnets cured in the presence of constant level of co-agents—30 phr. The content of magnetic filler was also kept constant in all rubber composites—50 phr, while dicumyl peroxide was dosed to the rubber formulations in concentration scale ranging from 1 to 10 phr. With increasing amount of DCP, more free radicals are formed in the elastomer matrix after thermal decomposition of the peroxide at a vulcanization temperature.

#### 3.2.1. Microscopic Analysis 

**Figure 12:**
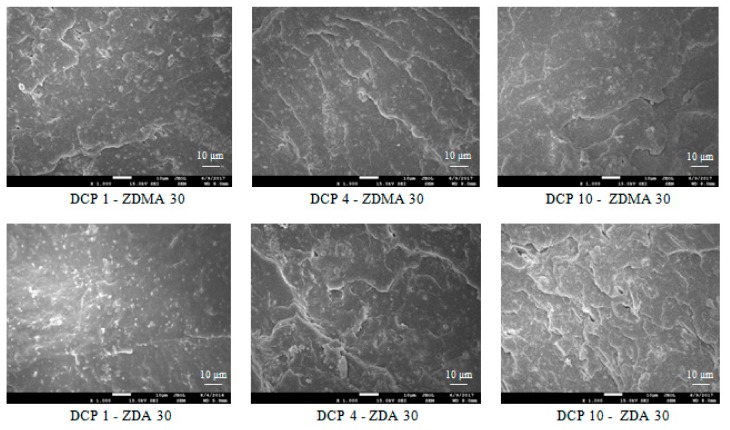
SEM images of composites cured with different content of peroxide.

From Figure 12, it is possible to observe that the dimensions of co-agents structures decrease with increasing amount of peroxide. Thus, it becomes evident that with more free radicals, more monomers from co-agents take part in the in-situ polymerization, resulting in the decrease of size and molecular weight of polymerized molecules of co-agents. 

#### 3.2.2. Cross-Link Density, Physical-Mechanical Properties, Dynamic and Magnetic Characteristics

**Figure 13:**
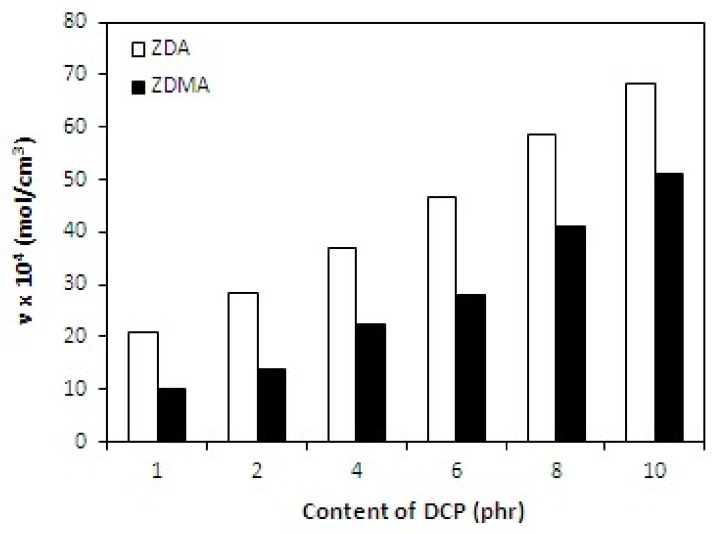
Influence of peroxide content on cross-link density *ν* of composites.

**Figure 14:**
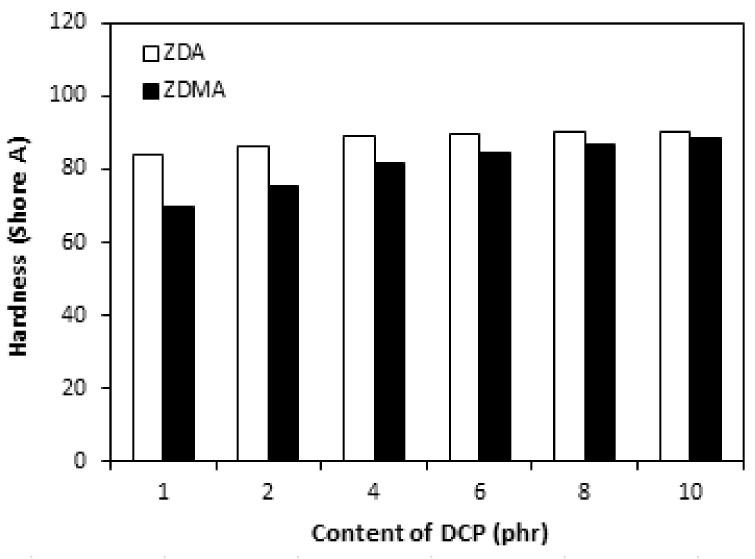
Influence of peroxide content on hardness of composites.

**Figure 15:**
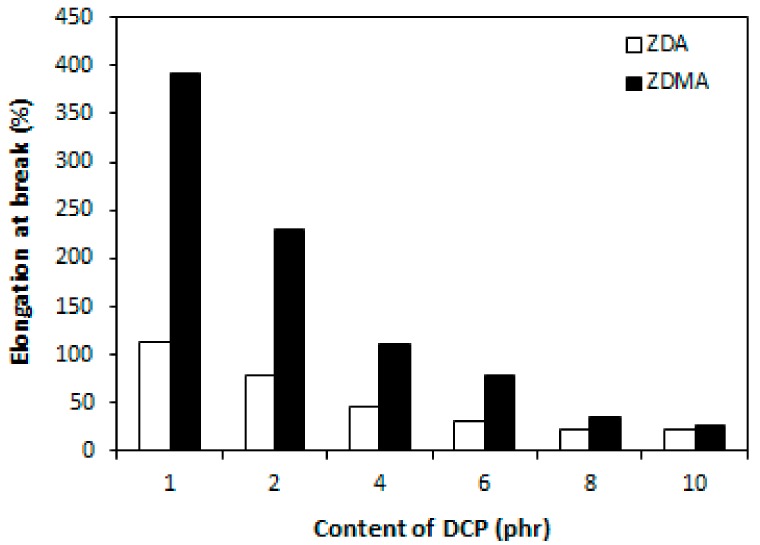
Influence of peroxide content on elongation at break of composites.

**Figure 16:**
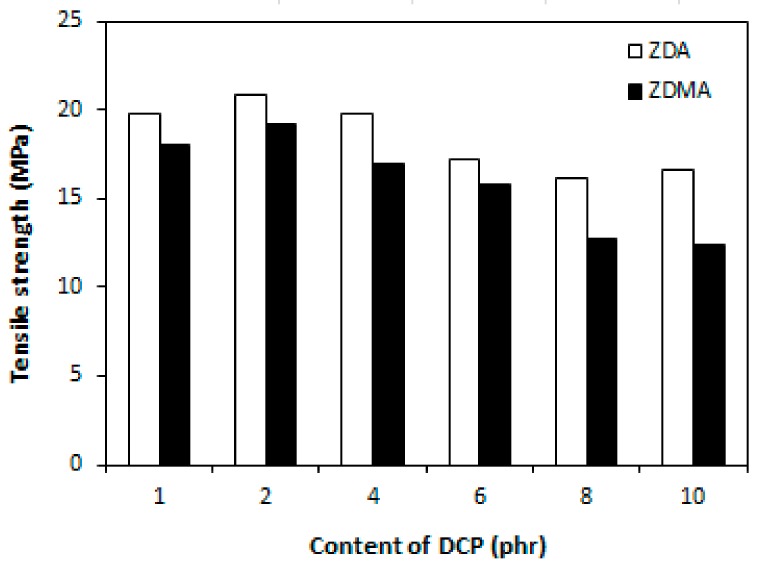
Influence of peroxide content on tensile strength of composites.

The cross-link density of rubber magnets showed a significant increasing trend with increasing amount of DCP (Figure 13). The higher was the amount of peroxide, the more free radicals derived from peroxide could participate in formation of cross-links between rubber chains. On the other hand, as already mentioned, peroxide free radicals could support polymerization of co-agent molecules into nano-structures and ionic clusters and their couplings onto rubber chains, which could contribute to the increase of cross-link density as well. The dependences of hardness and elongation at break followed the dependences of cross-link density, revealing that higher cross-link density of composites cured in the presence of DCP and ZDA was reflected in higher hardness and lower elongation at break of the equivalent composites (Figure 14 and Figure 15). The tensile strength of both type composites first slightly increased when the amount of peroxide increased up to 2 phr, then a decreasing trend of tensile strength was recorded with increasing content of peroxide (Figure 16). The reason can be attributed to a very high cross-linking degree. When the cross-link density is too high, the formed cross-links cause increased deformation stiffness in the rubber matrix, because of less mobility of rubber chains, which subsequently leads to the deterioration of tensile strength [51]. 

**Figure 17:**
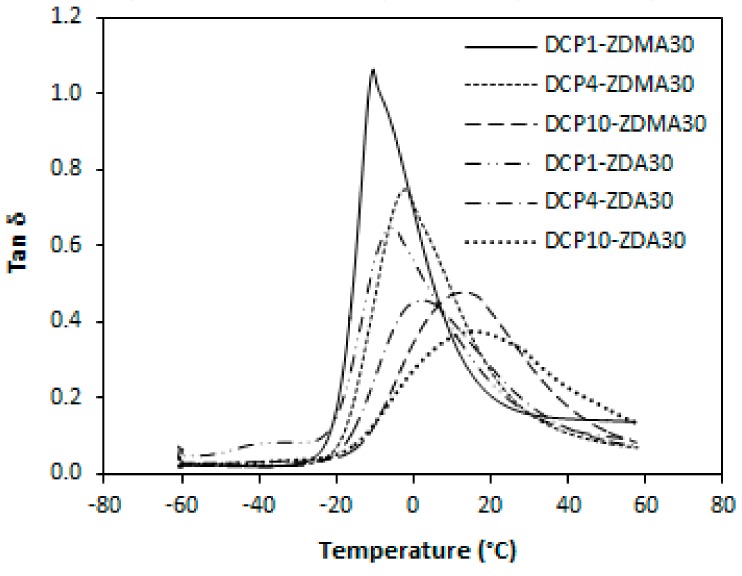
Variations of tan δ of composites cured with different content of peroxide.

The dynamic-mechanical analysis also confirmed a very close connection between cross-link density and glass transition temperature of composites. The higher was the cross-link density, the higher was the T_g_ (Figure 17, Table 5). Again, lower T_g_ showed composites that were cured with DCP and ZDMA with lower degree of cross-linking when compared to the equivalent composites cured with DCP and ZDA. It also becomes obvious from Table 5 that, in some cases, the T_g_ shifted over zero degree Celsius, especially in the case of composites cured with maximum amount of DCP, which is imputed to a significant restriction of rubber chains mobility due to a very high degree of cross-linking. 

**Figure 18:**
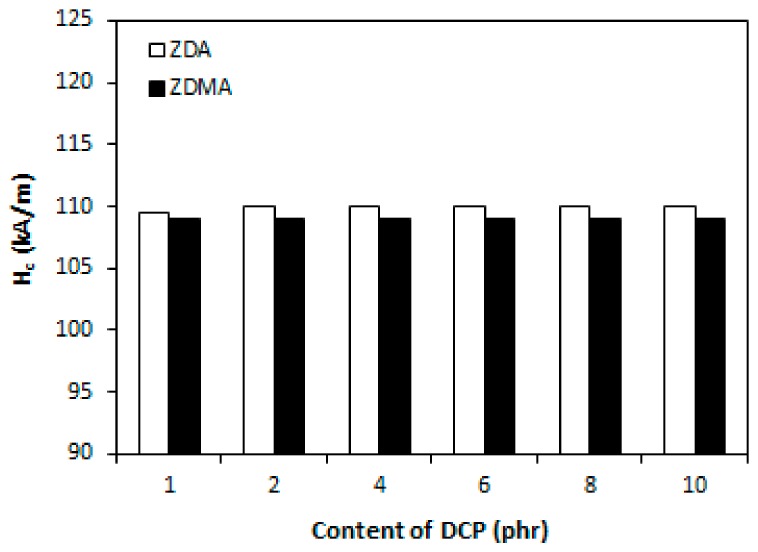
Influence of peroxide content on coercivity *H_c_* of composites.

**Figure 19:**
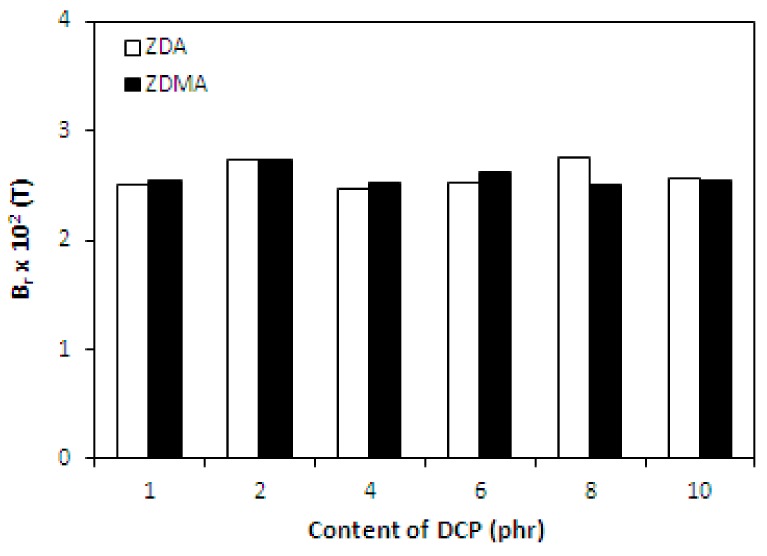
Influence of peroxide content on remanent magnetic induction *B_r_* of composites.

Finally, as seen in Figure 18 and Figure 19, the magnetic characteristics of composites were found not to be influenced by the amount of peroxide as their values moved in a close experimental range. The type of co-agent has also almost no influence on the magnetic properties of tested composites. The results just confirmed the presumption that magnetic characteristics of rubber magnets are dependent mainly on the type and content of applied magnetic filler with almost no influence of curing system composition, as it has also been demonstrated in our previous research [52,53,54].

## 4. Conclusions

NBR was compounded with strontium ferrite in order to prepare rubber magnets. Peroxide curing system, applied for cross-linking of the rubber magnetic composites, consisted of dicumyl peroxide as curing agent and zinc salts of acrylic and methacrylic acids as co-agents. The main goal was to examine the influence of the peroxide curing system on the cross-link density and properties of tested composites filled with constant level of magnetic filler.

The results showed that cross-linking and properties of peroxide cured rubber magnetic composites can be effectively improved by using of zinc acrylate and zinc methacrylate. Zinc based co-agents form complex network cross-link structure within the rubber matrix on one hand and they improve the adhesion to applied strontium ferrite on the other hand. Thus, they enhance the compatibility and adhesion between the filler and the rubber on the interphase which subsequently leads to the increase of the strength of composites. The dependences of physical-mechanical and dynamic properties were in close correlation with the cross-link density, demonstrating that the higher was cross-link density, the higher was modulus, hardness, and glass transition temperature of rubber magnets and the lower was elongation at break. Almost no changes in the magnetic characteristics of composites were recorded in dependence on the change in composition of curing systems applied. 

## Figures and Tables

**Table 1:** Characteristics of strontium ferrite.

Characteristics	Value
Total porosity (%)	54.94
Specific surface area (m^2^/g)	3.30
Total volume of pores (cm^3^/g)	0.264
Density (g/cm^3^)	4.13
Remanent magnetic induction (T)	0.127
Coercivity (kA/m)	116

**Table 2:** Composition of rubber compounds with different amount of co-agents.

Component	NBR	Ferrite	DCP	ZDA or ZDMA
**Content (phr)**	100	50	1	0–50

**Table 3:** Composition of rubber compounds with different amount of dicumyl peroxide.

Component	NBR	Ferrite	DCP	ZDA or ZDMA
**Content (phr)**	100	50	1–10	30

**Table 4:** Dependences of glass transition temperature T_g_ and cross-link density *ν* of composites on the amount of co-agents.

Co-Agent Content (phr)	*ν* × 10^4^ (mol/cm^3^)	T_g_ (°C)	*ν* × 10^4^ (mol/cm^3^)	T_g_ (°C)
	ZDMA		ZDA	
0	4.7	−12.3	4.7	−12.3
10	6.8	−12.1	10.5	−11.7
30	10.0	−10.4	20.9	−5.7
50	14.5	−7.3	23.7	−4.4

**Table 5:** Dependences of glass transition temperature T_g_ and cross-link density *ν* of composites on the amount of dicumyl peroxide.

Peroxide Content (phr)	*ν* × 10^4^ (mol/cm^3^)	T_g_ (°C)	*ν* × 10^4^ (mol/cm^3^)	T_g_ (°C)
	ZDMA 30 phr		ZDA 30 phr	
1	10.0	−10.4	20.9	−5.7
4	22.3	−2.2	37.1	2.1
10	51.3	13.1	68.3	15.2
